# Ensuring that offsets and other internationally transferred mitigation outcomes contribute effectively to limiting global warming

**DOI:** 10.1088/1748-9326/abfcf9

**Published:** 2021-06-23

**Authors:** Myles Allen, Katsumasa Tanaka, Adrian Macey, Michelle Cain, Stuart Jenkins, John Lynch, Matthew Smith

**Affiliations:** 1 Environmental Change Institute, School of Geography and the Environment, University of Oxford, Oxford, United Kingdom; 2 Atmospheric, Oceanic and Planetary Physics, Department of Physics, University of Oxford, Oxford, United Kingdom; 3 Laboratoire des Sciences du Climat et de l’Environnement (LSCE), IPSL, CEA/CNRS/UVSQ, Université Paris-Saclay, Gif-sur-Yvette, France; 4 Earth System Risk Analysis Section, Earth System Division, National Institute for Environmental Studies (NIES), Tsukuba, Japan; 5 Institute for Governance and Policy Studies, Victoria University of Wellington, Wellington, New Zealand; 6 Centre for Environmental and Agricultural Informatics, Cranfield University, Bedford, United Kingdom

**Keywords:** offsets, Paris Agreement, internationally transferred mitigation outcomes, voluntary carbon markets, greenhouse gas metrics

## Abstract

Ensuring the environmental integrity of internationally transferred mitigation outcomes, whether through offset arrangements, a market mechanism or non-market approaches, is a priority for the implementation of Article 6 of the Paris Agreement. Any conventional transferred mitigation outcome, such as an offset agreement, that involves exchanging greenhouse gases with different lifetimes can increase global warming on some timescales. We show that a simple ‘do no harm’ principle regarding the choice of metrics to use in such transactions can be used to guard against this, noting that it may also be applicable in other contexts such as voluntary and compliance carbon markets. We also show that both approximate and exact ‘warming equivalent’ exchanges are possible, but present challenges of implementation in any conventional market. Warming-equivalent emissions may, however, be useful in formulating warming budgets in a two-basket approach to mitigation and in reporting contributions to warming in the context of the global stocktake.

## 1. Background

Article 6 of the Paris Agreement provides for parties to help achieve their nationally determined contributions (NDCs) through internationally transferred mitigation outcomes (ITMOs). These may take several forms: ‘cooperative approaches’ (Article 6.2) such as the recent Switzerland–Peru agreement [1]; the market mechanism established under Article 6.4 but not yet operational; and non-market approaches (Article 6.8) for which a not-yet-operational ‘framework’ has been established. Common to all three is a party (or non-state actor) discharging an undertaking to reduce emissions by paying for or otherwise facilitating corresponding reductions of net emissions (including removals) by another party. ITMOs were extensively discussed at COP 25 in Madrid, 2019, and much remains unresolved [2].

The concerns about environmental integrity under Article 6 are sourced in the well-documented experience of the Kyoto Protocol’s flexibility mechanisms—international emissions trading, joint implementation and the clean development mechanism (CDM). Three major concerns are: use of ‘hot air’ to meet obligations, lack of additionality (where emissions reductions would have happened under business as usual and so create no increase in overall mitigation) and perverse incentives (e.g. HFC 23 destruction projects under the CDM which led the EU, New Zealand and other countries to ban units from these projects from their emissions trading schemes). Such concerns explain the cautious approach [3] many parties, and especially developing countries, are taking to Article 6, which is effectively replacing the Kyoto mechanisms but in a broader context where all countries will be undertaking mitigation contributions via their NDCs. Here we focus specifically on the challenge of ensuring the environmental integrity of transfers that involve multiple greenhouse gases (GHGs), and in particular how to avoid unintended warming outcomes resulting from such transfers involving GHGs of different atmospheric residence times.

The use of ‘robust accounting’ to help ensure transparency and environmental integrity is a requirement of Article 6. Three possible definitions of environmental integrity have been identified [4] in the context of Article 6: aggregate achievement of mitigation targets; no increase in global aggregate emissions; and a decrease of global aggregate emissions. All present challenges in the context of multi-gas trading. The 2nd and 3rd definitions both depend on the metric used to aggregate emissions as well as on the counterfactual case in the absence of trading, while the first needs to be qualified ‘where these targets support the achievement of the long-term temperature goal (LTTG)’ (many current ‘mitigation targets’ represent increases of emissions above what would be expected without further policy intervention, so simply meeting and not exceeding these is clearly inconsistent with the LTTG [5]). In the context of the Paris agreement, however, mitigation is undertaken explicitly ‘in order to achieve’ the LTTG, so any outcome or mitigation instrument, such as an ITMO, that might compromise the achievement of the LTTG could be seen as compromising environmental integrity.

While discussion of accounting metrics is continuing under the UNFCCC, it was agreed at COP24 in Katowice that parties would use 100 years time-horizon global warming potential (GWP_100_) values from the IPCC 5th Assessment Report [6] (AR5) to report aggregate emissions and removals of GHGs, expressed as CO_2_-equivalent. The adoption of consistent GWP values is welcome, and provided net emissions of individual gases are also reported separately, which is also required by the UNFCCC reporting protocols, it does not compromise transparency.

## 2. Problems with the environmental integrity of multi-gas transactions

**Figure 1.**
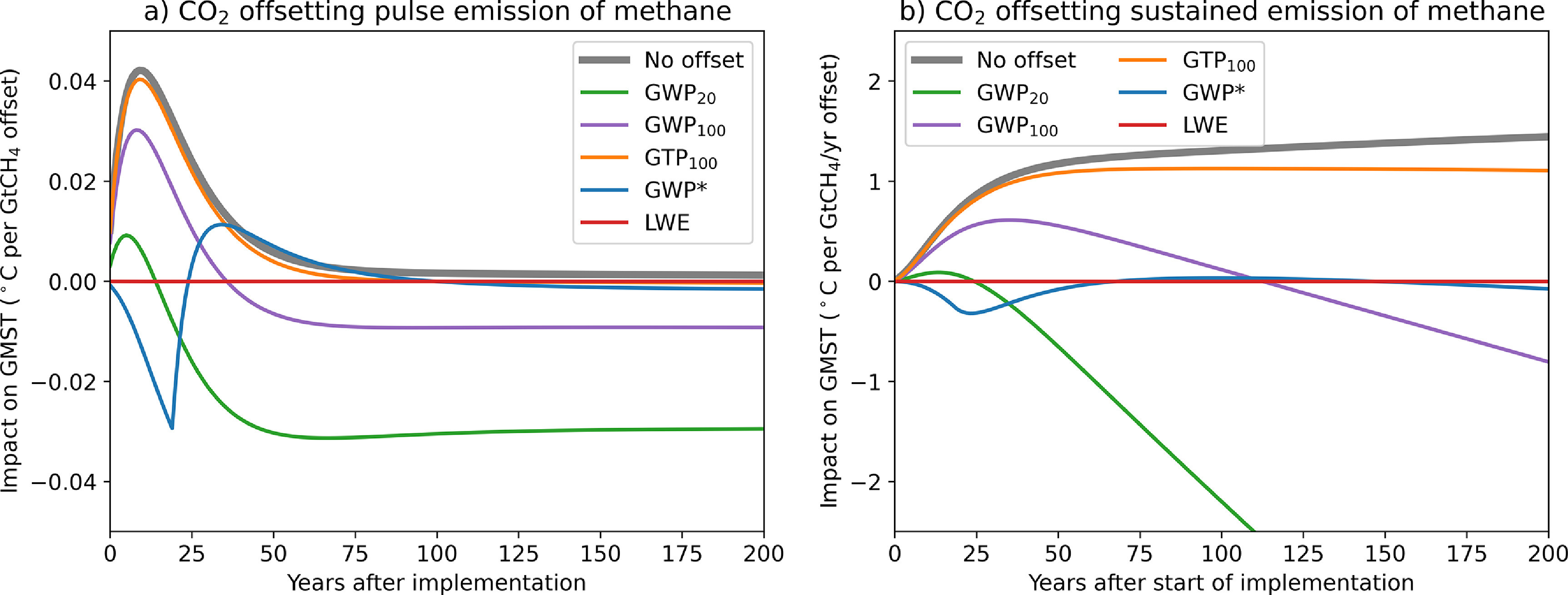
Impact on global mean surface temperature of transfers involving ‘offsetting’ the emission of methane with avoided emission or removal of CO_2_. Left panel shows the impact of a one-off transfer occurring in year 0, while the right panel shows the impact of a sustained transfer offsetting a constant rate of emission of methane with a constant rate of avoided emission or removal of CO_2_, starting in year 0. Green, purple and yellow lines show impact on global temperature when the amount of CO_2_ is calculated using GWP_20_, GWP_100_, & GTP_100_, respectively, blue lines using warming-equivalent emissions calculated using GWP* and red lines using linear warming-equivalent emissions. Grey lines show warming caused by methane emissions without any CO_2_ offsetting. Based on the ‘do no harm’ principle proposed here, GWP_20_ would be the recommended conventional metric for this class of transaction. All calculations performed using the standard AR5 impulse response model with thermal response parameters scaled to give an equilibrium climate sensitivity of 2.75 °C (original model was 3.9 °C) [6].

**Figure 2.**
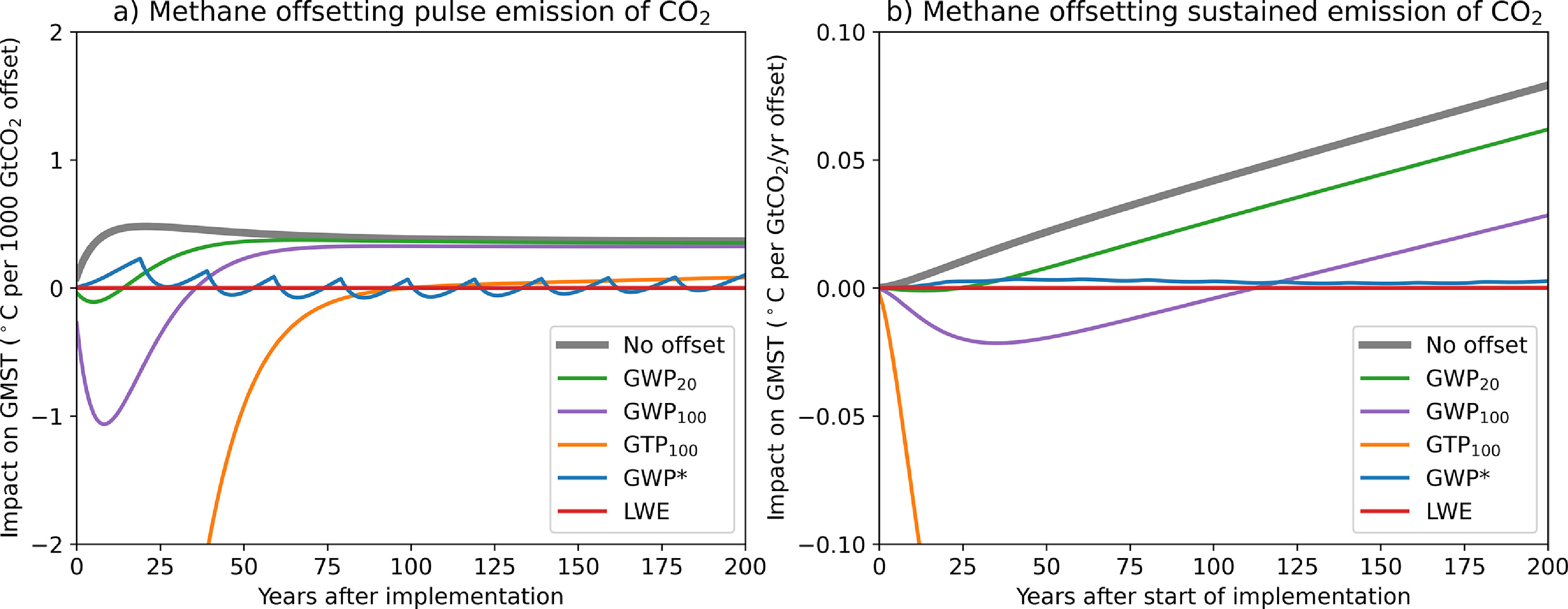
As figure 1, but for transfers involving offsetting emission of CO_2_ with avoided emission of methane. GTP_100_ would be the recommended conventional metric for this class of transaction under a ‘do no harm’ principle.

Relying exclusively on GWP_100_ in ITMOs or offset transactions, however, could increase global warming on some timescales, contrary to the overall aim of Article 2 of the Paris Agreement which sets out to limit warming and does not specify a timescale. For example, suppose a party or non-state actor A decides to emit 1 t CO_2_-equivalent of methane, a potent but short-lived climate pollutant (SLCP), that they had otherwise pledged to avoid emitting. Instead, A decides to pay B to sequester 1 t CO_2_-equivalent of a very-long-lived, cumulative pollutant like CO_2_. Although it has no impact on nominal aggregate CO_2_-equivalent emissions calculated using GWP_100_, this transaction results in an increase in global temperature for approximately 45 years, and lowered temperatures thereafter (purple line in figure 1(a)). If, conversely, A decides to offset the emission of 1 t of CO_2_ by paying B to avoid emitting 1 t CO_2_-equivalent of methane, global temperatures are increased on all timescales greater than 45 years (purple line in figure 2(a)) [7–9].

Given the current level and rate of warming (1.2 °C and about 0.25 °C per decade respectively [10]), any scenario that limits warming to ‘well below 2 °C’ must require, by simple geometry [11], a substantial slow-down if not a complete halt to warming by 2060. Hence any transaction that results in an increase in warming for 45 years, or any timescale on which temperatures might peak, risks compromising the achievement of the LTTG and hence environmental integrity. Likewise, the Paris Agreement did not set out only to limit warming by mid-century without regard to what happens thereafter, so a transaction that increases global temperatures after 2060 could also be argued to be inconsistent with the LTTG.

Replacing GWP_100_ with some other metric, such as the 20-year GWP_20_, or 100-year global temperature-change potential, GTP_100_, does not solve this problem, since either one transaction or the other would inevitably result in an increase in global temperature on some timescale. The effect is even more pronounced when considering the impact of offsetting sustained emissions. Using avoided methane emissions, landfill methane capture and destruction or restoring tides to coastal wetlands [12] to offset sustained CO_2_ emissions using GWP_20_ (green line in figure 2(b)) would cause temperatures to increase continuously from year 30 onwards, while using GTP_100_ to offset sustained methane emissions with CO_2_ removal causes immediate substantial warming (yellow line in figure 1(b)).

## 3. A dual valuation proposal

Since it is not known when peak warming will occur, any instrument that results in higher global temperatures on any timescale risks compromising the achievement of the LTTG. It has been argued [13] that, because of the challenge of limiting warming to 1.5 °C, ‘pursuing efforts’ should be interpreted as a commitment to return temperatures to below 1.5 °C by 2100, hence providing a timescale. Article 2 of the Paris Agreement is, however, more commonly [14] interpreted as a single goal requiring parties to hold global temperatures ‘well-below 2° C’ and as close to 1.5 °C as they can. Moreover, many adverse impacts of climate change, and hence the risk of dangerous anthropogenic interference in the climate system, increase with peak warming [15] even if temperatures decline thereafter. Hence any instrument, such as a CO_2_-for-methane exchange denominated in GWP_100_, that increases peak warming further above 1.5 °C, or increases the risk of peak temperatures exceeding 2 °C, is difficult to reconcile with the fundamental aims of both the Paris Agreement and the UNFCCC itself.

To guard against this unintended outcome, parties to any ITMO or offset contract could use a metric value among those assessed by the IPCC that results in ‘an overall mitigation of global emissions’ [16] whichever metric is used to calculate it. Given the results in figures 1 and 2, this would ensure that the transaction does not significantly increase global warming on any policy-relevant timescale, consistent with the spirit of Article 6.4: throughout the agreement it is clear that mitigation is undertaken ‘in order to meet the LTTG’.

Applying this principle would mean using GTP_100_ to calculate the amount of avoided methane emissions required to offset the emission of CO_2_ (yellow lines in figure 2), and using GWP_20_ to calculate the avoided CO_2_ emissions or CO_2_ sequestration required to offset the emission of methane (green lines in figure 1). If a cumulative pollutant is being used to offset the emission of a SLCP, the risk is that this might cause short-term warming, so a metric reflecting short-term behaviour such as GWP_20_ is used. Conversely, if a SLCP is being used to offset the emission of a cumulative pollutant, the risk is that this might cause warming in the long term, so a metric that reflects long-term behaviour like GTP_100_ is used.

The use of GWP_20_ and GTP_100_ as bounding valuations is somewhat arbitrary: why not GTP_75_? We suggest these because there is some familiarity with them in both the IPCC and UNFCCC, but the concept of warming-equivalent emissions, discussed further below, provides a less arbitrary justification for a broadly similar range of values.

This ‘dual valuation’ proposal is inspired by the concept of ‘dual accounting’ [17], extended to GTP_100_ to avoid over-representing the short-term response [18]. Reference [17] argue that GHGs should be reported using at least two metrics to emphasise the distinct timeframes of their impacts, but leave open the question of which metric should be used in any individual decision or transaction. Our proposal extends this using a transparent ‘do no harm’ (on any policy-relevant timescale) decision rule.

The broad spread between ‘buying’ and ‘selling’ valuations might discourage exchanges involving gases with very different lifetimes. While this could hamper net progress towards mitigation targets due to higher costs for GHG abatements as a result of the restricted use of ITMOs, it would also discourage ‘lock-in’ of policies involving unsustainable combinations of emissions and removals [19]. This reflects previous calls for a ‘two-basket’ approach to mitigation, where it has been argued that shorter- and longer-lived gases are best constrained under separate policies [9]. It would also support any stocktake of progress towards a LTTG: it is impossible to assess the impact on global temperatures of emissions pledges expressed as CO_2_-equivalent emissions aggregated using any pulse-emission metric (so including GWP_20_, GWP_100_ and GTP_100_) involving an unspecified mix of long-lived and short-lived GHGs.

The use of dual valuation in ITMOs would ensure that overall warming on all timescales is either the same as or lower than would occur in the absence of any transferred mitigation outcomes. Hence, if a global stocktake of aggregate contributions to mitigation outcomes without transfers were consistent with achieving a LTTG, then if transfers are allowed using dual valuation and (an important proviso) issues with additionality and avoidance of double-counting are addressed, then they would also be consistent with achieving that LTTG with transfers. There are, however, more fundamental problems, that we do not address here, in how ITMOs are reflected in parties’ own NDCs. These issues arise under any regime of participant-determined contributions, and remain under discussion [20].

Allowing ITMOs with dual valuation could, in principle, improve economic efficiency over a strict two-basket approach without compromising environmental integrity. Under a two-basket approach, the amount of mitigation of short-lived versus long-lived GHGs has to be set by policy rather than discovered by the market, which could conflict with the cost-effectiveness principle of the UNFCCC (Article 3.3). Many marginal abatement cost curves for SLCPs are, however, strongly non-linear [21], with a large fraction of emissions avoidable at very low cost. In principle, there is an economic efficiency argument for allowing the market to discover these opportunities, but because they are so low-cost, they may be expected to occur independent of how ITMOs are defined. The advantage of dual valuation is that it ensures these reductions can still occur, but are not over-valued in terms of CO_2_, thus minimising the degree to which they undermine incentives for CO_2_ emissions reductions.

## 4. Climate neutral transactions using warming-equivalent emissions

To illustrate the difficulties inherent in transactions involving gases with very different lifetimes, we consider what it would take to make such transactions genuinely ‘climate neutral’, in the sense of not causing warming or cooling on any timescale. This would require formulating ITMOs and offsets in terms of ‘warming-equivalent’ emissions.

Methods exist that have been designed to find emissions of SLCPs that approximate the impact of CO_2_ emissions on global temperatures on all timescales, and could therefore be used to explore climate neutrality [22, 23]. Various formulations of warming-equivalent emissions have been proposed, either explicitly or implicitly [8, 24, 25], and although they differ in details, they share the common feature that a pulse emission of CO_2_ is considered approximately equivalent to a permanently sustained change in the emission rate of methane or any SLCP.

The blue lines in figures 1 and 2 show the impact of one recently-proposed [22] method of calculating warming-equivalent emissions, GWP*, which uses a ‘flow’ term to represent the short-term impact of any change in SCLP emission rate, and a ‘stock’ term to represent the longer-term adjustment to past increases (the original GWP* formulation [26] simply equated a one-off pulse CO_2_ emission with a sustained increase in SLCP emission rate). Coefficients are further refined to be precisely consistent with radiative forcing from the AR5 impulse response model (see section 6, and [27] for the full derivation).

**Figure 3.**
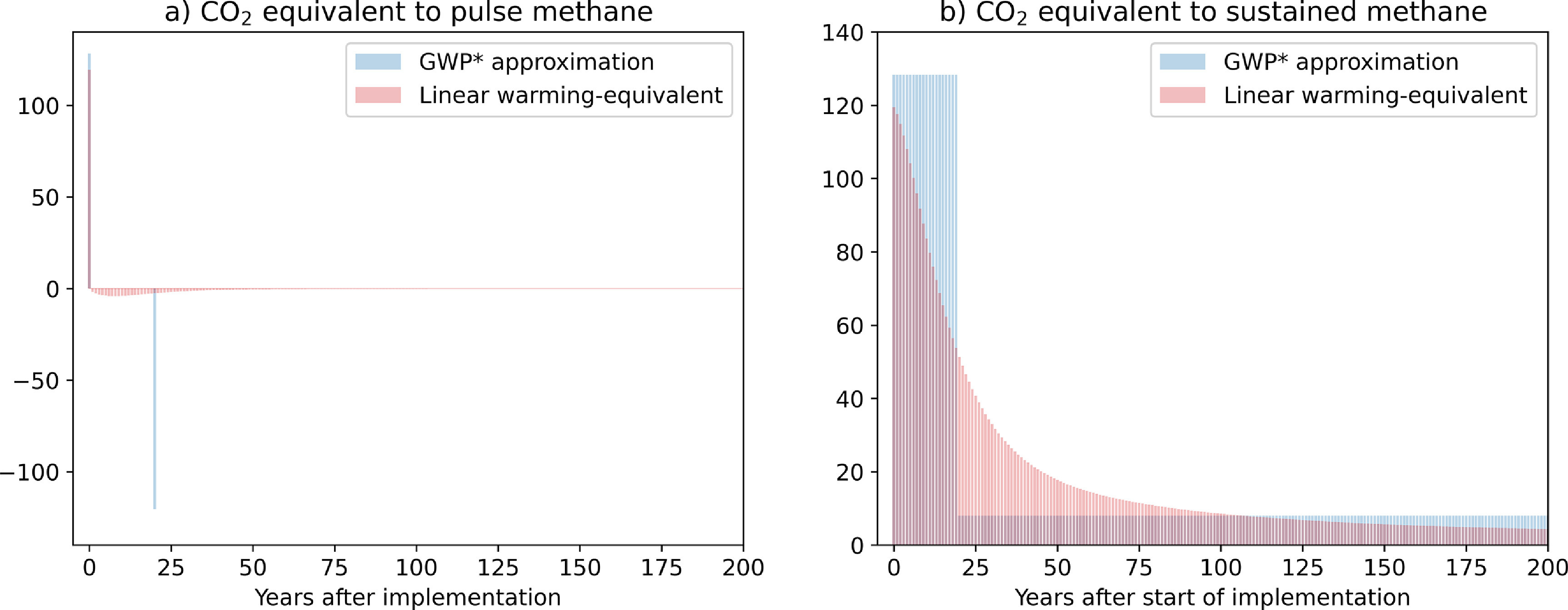
Warming-equivalent emissions of CO_2_ giving the same forcing response to (a) a pulse emission of methane in year 0 and (b) a sustained constant emission of methane starting in year 0, calculated using the GWP* approximation in blue and exact linear warming equivalent (LWE) emissions (multiplying the forcing response to methane emissions by the inverse of the CO_2_ absolute global forcing potential (AGFP) matrix—see section 6) in red.

**Figure 4.**
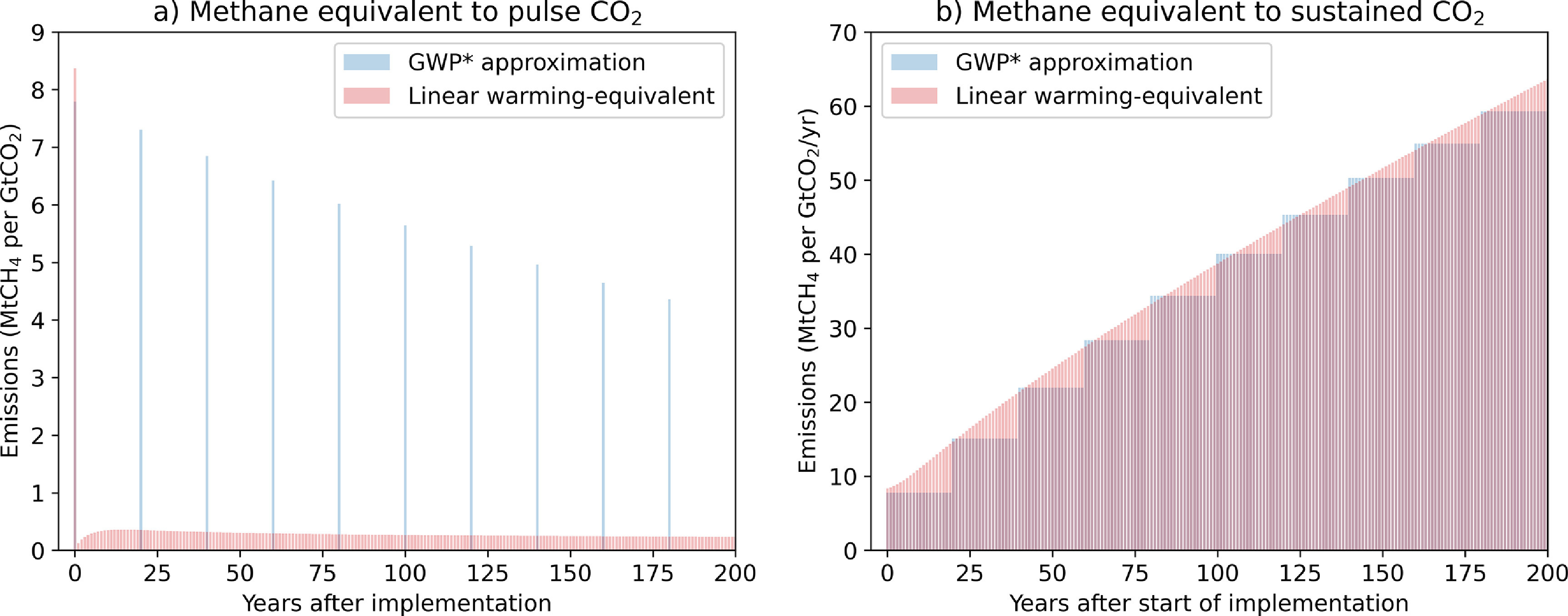
Warming-equivalent emissions of methane giving the same forcing response to (a) a pulse emission of CO_2_ in year 0 and (b) a sustained constant emission of CO_2_ starting in year 0, calculated using the GWP* approximation in blue and exact LWE emissions in red.

This method equates an 1 t yr^−1^ increase in methane emission rate (1 tCH_4_ yr^−1^) with an emission of 128 tCO_2_ yr^−1^ for the 20 years after the increase occurs, followed by 8 tCO_2_ yr^−1^ thereafter (figure 3(b)). The AR5 value of GWP_100_ for methane (28) is reflected in these coefficients: warming-equivalent emissions ${E^ * }\left( t \right) = 4.53E\left( t \right) - 4.25E\left( {t - 20} \right)$ for any SLCP, where $E\left( t \right)$ are CO_2_-equivalent emissions calculated using GWP_100_, hence ${E^*}\left( t \right)$ is easily calculated for any SLCP reported under UNFCCC guidelines. They capture both the large immediate warming impact of any increase in methane emission rates, and the much lower warming impact of sustained methane emissions [28]. Under GWP*, a pulse emission of methane is equated with an immediate pulse emission of CO_2_ followed by a slightly smaller pulse CO_2_ removal 20 years later (figure 3(a)), while a pulse emission of CO_2_ is equated with ongoing methane emissions represented by a succession of methane pulses declining exponentially in magnitude (see section 6 and figure 4(a)). Hence a warming-equivalent offset of either gas involves an immediate removal (or avoided emissions) of the other gas plus a commitment to further emissions or removals in the future.

Although GWP* is an improvement on any of the non-warming-equivalent metrics, particularly when applied to the offsetting of sustained emissions of either CO_2_ or methane (blue lines in figures 1(b) and 2(b)), we can go one stage further, and calculate the ‘Linear Warming Equivalent (LWE)’ methane emissions required to compensate exactly for the warming caused by a CO_2_ emission and vice versa by inverting the linear impulse-response model used to evaluate metric values (see section 6). This calculation, which is both exact and metric-independent (since the same model is used for all metrics), implies that a pulse emission of 1 tCH_4_ has the same warming impact as a pulse emission of 120 tCO_2_ (the ratio of methane, including indirect effects, to CO_2_ radiative efficiencies per tonne [6]) followed by sustained CO_2_ removal following a continuously-varying profile that removes an average of 2 tCO_2_yr^−1^ for the first 50 years, and declines thereafter (figure 3(a), red). A pulse emission of 1000 tCO_2_ has the same warming impact as a pulse emission of 8.4 tCH_4_ followed by sustained methane emission at an average rate of 0.32 tCH_4_ yr^−1^ for the first 50 years and declining thereafter (figure 4(a), red). Transactions based on LWE emissions have, by construction, no impact on global temperature on any timescale (subject to the linearisation underlying the impulse-response model), shown by the red lines in figures 1 and 2.

Comparing red and blue emissions series in figures 3(a) and (b) suggests the GWP* metric might be further improved by defining the change in methane emission rate as the difference between the current years’ emissions and average emissions over the past 40 years, rather than the instantaneous value 20 years ago. This is indeed the case, and also has the advantage of reducing the dependency of current GWP* emissions on events that occurred 20 years ago. Since, however, this complicates the definition of GWP* and has no impact on cumulative GWP* emissions on multi-decade timescales, we continue to use the published formulation here.

There is no geophysical reason why warming-equivalent emissions could not be used in the formulation of fully climate neutral offsetting contracts and ITMOs. There are, however, evident challenges [13] in implementing warming-equivalent exchanges, in particular in a party or non-state actor taking on an obligation to an indefinitely-sustained commitment to avoided emissions in future, as would be the case if SLCPs are used to offset CO_2_ emissions [9, 29]. Such commitments become particularly problematic at a time when the supply of emissions to be avoided is declining because of global mitigation efforts. As a thought experiment, an alternative to indefinite commitments would be to agree a set time-frame for avoided SLCP emissions, with the remaining balance offset by a one-off CO_2_ removal: for example, if methane were to offset a pulse emission of 1000 GtCO_2_, near-exact warming equivalence could be obtained with an immediate removal or avoided emission of 1000/128 = 7.8 tCH_4_ followed by a removal of 938 (1000 × 120/128) tCO_2_ after 20 years, when the next pulse of methane ‘comes due’ in figure 4(a).

These climate-neutral transactions formulated in terms of warming-equivalent emissions also explain why the apparently ad-hoc proposal in the first part of this paper works as it does: when CO_2_ removal is being used to offset methane emissions, we need a removal of order 100 tCO_2_/tCH_4_ to match the immediate impact of a methane emissions pulse shown in figure 3(a), even though much of that CO_2_ could, in a perfect warming-equivalent transaction, be reemitted over the following decades. Hence an exchange rate comparable to GWP_20_ must be used to avoid a short-term warming. In contrast, when avoided methane emissions are being used to offset CO_2_, a total of 1/8th tCH_4_/tCO_2_ needs to be eventually removed or avoided to compensate for a CO_2_ emission pulse (summing to infinity the blue geometric series in 4a), much more than the 1/28th or 1/84th tCH_4_ implied by GWP_100_ or GWP_20_, and closer to the rate implied by GTP_100_. This also corresponds to the 8:1 ratio required to offset a sustained emission of either gas that has been constant for at least 20 years (figure 3(b)).

**Figure 5.**
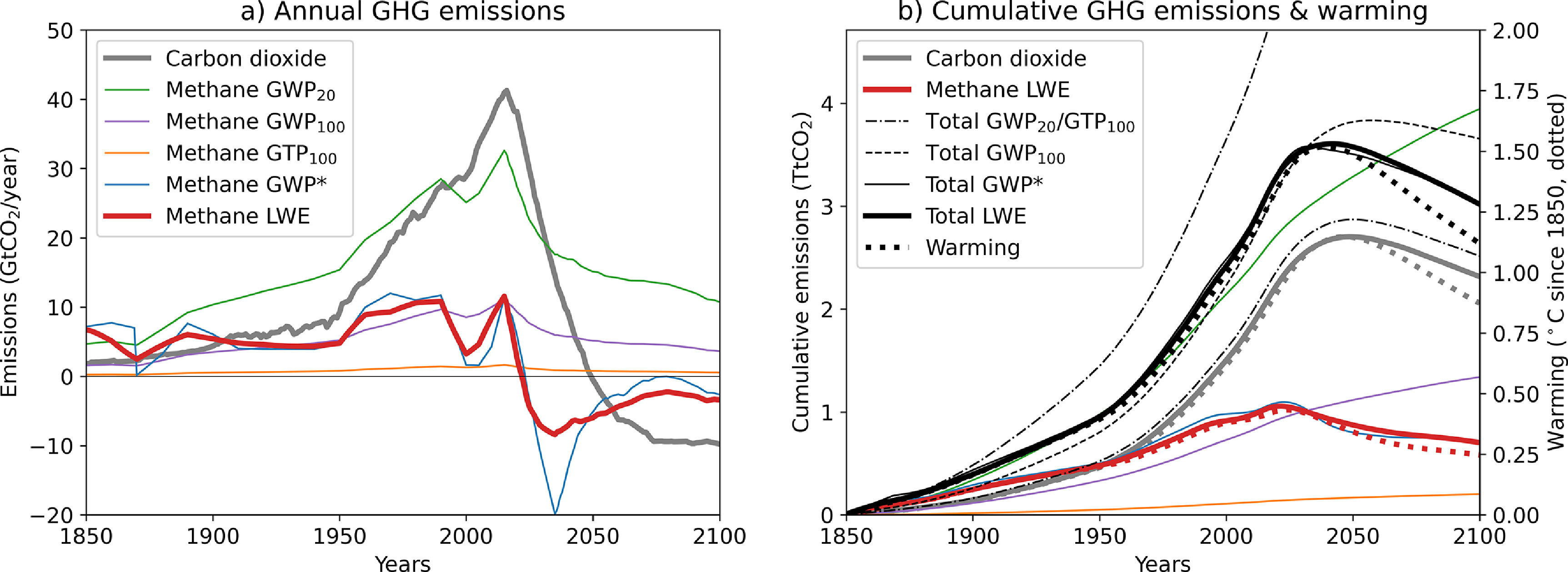
(a) Annual emissions of CO_2_ (grey) and methane (other colours) under various metrics for a representative 1.5 °C-consistent scenario. Thin lines show metric-equivalent methane emissions using GWP_100_ (purple), GWP_20_ (green) and GTP_100_ (yellow). Thick red line shows exact LWE emissions obtained by inverting the AR5 linear response model, while thin blue line shows the GWP* approximation. (b) CO_2_-induced (grey dotted), methane-induced (red dotted) and combined (black dotted) warming calculated with the AR5 linear impulse-response model compared with cumulative emissions under the various metrics. Cumulative totals from both gases are shown aggregated using GWP_100_ (dashed black), LWE (thick black), GWP* (thin black), GWP_20_ and GTP_100_ (upper and lower dash-dot black).

Finally, we re-emphasise how warming-equivalent emissions can be used to inform policies in a two-basket approach [9] to mitigation under a global temperature goal, by relating cumulative emissions directly to temperature outcomes [22]. CO_2_-warming-equivalent emissions have, by construction, approximately the same impact on global temperatures as CO_2_ emissions. Figure 5(a) shows annual emissions of CO_2_ and methane under a range of metrics for a representative 1.5 °C scenario (the median emissions profile of 1.5 °C scenarios in SR1.5 [30]), while figure 5(b) compares cumulative emissions under these different metrics with warming calculated with the AR5 linear model. Cumulative emissions of CO_2_ and both exact (LWE) and approximate (GWP*) warming-equivalent emissions of methane match CO_2_-induced, methane-induced and combined warming up to the time of peak warming (and would match cooling trends after peak warming if compared to a non-linear model that accounts for changing airborne fraction [11, 29]). This is a linear calculation, and hence can be used to assess both historical contributions to warming and contributions to achieving a temperature goal for individual countries and non-state actors. In contrast, cumulative CO_2_-equivalent emissions of methane aggregated using the conventional GWP_100_ are effectively meaningless: they happen, by coincidence, to be approximately proportional to methane-induced warming to date, but diverge as soon as methane emissions start to fall, while cumulative CO_2_-equivalent methane emissions under both GWP_20_ and GTP_100_ fail to reflect historical contributions to warming entirely.

## 5. Conclusions

There are many challenges in the effective implementation of ITMOs and offset markets, including monitoring, verification, double-counting, additionality and permanence [31]. For ITMOs or offset contracts to cause global warming by design, however, is both undesirable and avoidable. Our ‘dual valuation’ proposal, valuing transactions using the emission metric that results in an overall mitigation of global emissions whatever metric is used to evaluate it, would represent a simple way to take advantage of some opportunities for low-cost SLCP emission reductions without compromising the overall aim of the Paris Agreement to limit the increase in global average temperatures (with no specified timescale). It is consistent with both the underlying scientific framework and metrics presented in AR5 (which informed the Paris Agreement), and more recent research on alternative metric concepts. More work is needed to determine whether insisting on climate neutrality or better in ITMOs using dual valuation would lead to an overall increase in climate mitigation.

A two-basket approach, under which emissions of cumulative pollutants and SLCPs are specified separately in inventories, NDCs and mid-century long-term strategies would be the most robust in terms of supporting stocktakes of progress to a LTTG, because there would then be a transparent link between reported and projected emissions and warming outcomes. But however desirable scientifically, the potential costs of a pure two-basket approach should also be recognised. Suppose country A is implementing an economy-wide carbon price of $25 per tCO_2_, while methane abatement opportunities are available in country B for less than $100 per tCH_4_ that are not being realised because country B has not adopted a particularly ambitious NDC. This is clearly inefficient on any measure. The simplest solution would be for country B to enhance the ambition of the SLCP component of its NDC, but this may take time, and require additional resources. In the meantime, introducing ITMOs using dual valuation would allow country A to support achieving those methane abatement opportunities without flooding the market and undermining their domestic CO_2_ mitigation efforts.

We also show that fully climate neutral transactions could be constructed, but if SLCPs are used to completely offset CO_2_ emissions, these would require a potentially indefinite commitment to future emission reductions or removals to compensate for the climate impact of current CO_2_ emissions, presenting even more implementation challenges. Either exact or approximate warming-equivalent emissions can, however, be used to compare the global temperature implications of separate targets for cumulative climate pollutants and SLCPs in a two-basket approach to mitigation in pursuit of a LTTG.

## 6. Methods

For methane with a GWP_100_ of 28.4 and using updated coefficients [27] for GWP*, CO_2_-warming-equivalent emissions are given by ${E^ * }\left( t \right) = 128 \times {E_{{\text{C}}{{\text{H}}_{\text{4}}}}}\left( t \right) - 120 \times {E_{{\text{C}}{{\text{H}}_{\text{4}}}}}\left( {t - 20} \right)$, where ${E_{{\text{C}}{{\text{H}}_{\text{4}}}}}\left( t \right)$ are methane emissions at time $t$, and ${E_{{\text{C}}{{\text{H}}_{\text{4}}}}}\left( {t - 20} \right)$ methane emissions in the 20 years earlier. CO_2_-warming-equivalent emissions corresponding to a 1 tCH_4_ pulse emission of methane in year 0 are therefore a pulse of 128 tCO_2_-we in year 0 and a pulse removal of 120 tCO_2_-we in year 20 (blue bars in figure 3(a)), as the two terms on the RHS of the definition become non-zero at these respective points in time. Coefficients from ref. [22] are scaled by a factor of 1.13 to ensure an exact match between 100 years integrated radiative forcing caused by a pulse methane emission and that caused by the warming-equivalent emission of CO_2_ [27]. This improves consistency with the underlying linear impulse response model and the modelled response to ambitious mitigation scenarios (as expected, because the impulse response model is tuned to a constant-composition scenario).

Methane warming-equivalent emissions under GWP* corresponding to a 1000 tCO_2_ pulse are a 1000/128 = 7.8 tCH_4_ pulse in year 0 (the first term on RHS of the definition of ${E^ * }$, because in this case ${E_{{\text{C}}{{\text{H}}_{\text{4}}}}}\left( {t - 20} \right) = 0$). After 20 years, ${E_{{\text{C}}{{\text{H}}_{\text{4}}}}}\left( {t - 20} \right) = 7.8$ tCH_4_, so to match the impact of ongoing zero emissions of CO_2_, a further emission of 7.8 × 120/128 = 7.3 tCH_4_ is required to give zero warming-equivalent emissions ${E^ * }$. This is followed by a sequence of pulses at 20-year intervals each 120/128 of the previous pulse (blue bars in figure 4(a)), giving an eventual total of (1000/128)/(1−120/128) = 125 tCH_4_, using the standard formula for summing a geometric series. Figures 3(b) and 4(b), for step emission profiles, are simply the time-integral of a series of the pulses shown in figures 3(a) and 4(a) respectively.

Exact LWE emissions can be calculated by noting that the forcing timeseries resulting from any emission perturbation timeseries of a GHG A, under the linearity assumptions inherent in all metric calculations, is given by the equation ${\mathbf f}= {{\mathbf F}_{\text{A}}}{{\mathbf e}_{\text{A}}}$ where the $i$th element of the vector ${\mathbf f}$ is the forcing in year $i$, the $j$th element of the vector ${{\mathbf e}_{\text{A}}}$ is emissions in year $j$, and ${{\mathbf F}_{\text{A}}}$ is a lower-diagonal Toeplitz matrix the first column of which is the first derivative of the AGWP of gas A, known as the Absolute Global Forcing Potential, AGFP [23]. The next column is identical to the first column lagged by 1 year and so on, so ${\left( {{{\mathbf F}_{\text{A}}}} \right)_{ij}} = {\text{AGF}}{{\text{P}}_{i - j + 1}} = {\text{AGW}}{{\text{P}}_{i - j + 1}} - {\text{AGW}}{{\text{P}}_{i - j}}$ for all $i \geqslant j$ and zero otherwise. Because the AGFP matrix is generally invertible, the emissions anomaly timeseries of gas B that gives an identical forcing history and hence temperature response to an emissions anomaly timeseries of gas A is given by ${{\mathbf e}_{\text{B}}} = {\mathbf F}_{\text{B}}^{ - 1}{{\mathbf F}_{\text{A}}}{{\mathbf e}_{\text{A}}}$.

Warming caused by a timeseries of CO_2_ emissions representing the exact LWE counterpart to a timeseries of methane emissions is identical to the warming caused by those methane emissions. Hence LWE emissions, by construction, indicate precisely the same sensitivity of warming at some arbitrary date in the future to variations in emissions now as is given by the time-dependent GTP [32]. Warming-equivalent emissions can thus be thought of as a generalisation of the time-dependent GTP from a single-year pulse to a complete emissions history.

Timeseries of CO_2_ emissions that give identical forcing and hence warming responses to pulse and constant methane emissions under the linear impulse response model used for metric calculations in AR5 are shown in red in figure 3, while figure 4 shows warming-equivalent emissions of methane corresponding to pulse and constant CO_2_ emissions. Thick red lines in figure 5 show annual and cumulative linear-warming-equivalent emissions of methane calculated by applying this formula to the full 251 years emissions timeseries 1850–2100. The operation clearly acts as a strong high-pass filter, equating strongly declining methane emissions with negative warming-equivalent emissions of CO_2_, as required to have the same impact on global temperatures.

Figure 3 also explains why it is important that a time-interval $\Delta t$ in the definition of GWP* must be of the order of 20 years: the size of the coefficients multiplying $E\left( t \right)$ and $E\left( {t - \Delta t} \right)$ are inversely proportional to this time-interval. If $\Delta t$ is substantially less that 20 years, then the coefficient multiplying $E\left( t \right)$ exceeds the ratio of the instantaneous radiative efficiencies of methane and CO_2_. This time-interval was presented in [22, 26] as a pragmatic choice, but it turns out to play a more fundamental role [27]. Confusion over this [33] has led to a widespread misconception that warming-equivalent emissions are only applicable to global scenarios. This cannot be the case because global emissions are simply the sum of contributions expressed in any linear metric, so warming-equivalent emissions can be calculated on any scale. The sensitivity to $\Delta t$ is simply less obvious for smoother global timeseries. On timescales shorter than 20 years, exact LWE emissions give a more accurate indication of warming-equivalent emissions but whether this precision is worth the additional complexity is debateable, since internal variability would mask the temperature response even to rapid forcing changes on these timescales.

## Data Availability

A Python notebook ITMO_figs.ipynb and datafile IAMC_med15_plushist.csv to reproduce the calculations and figures in this article are provided as supplementary files (available online at stacks.iop.org/ERL/16/074009/mmedia and at www.oxfordmartin.ox.ac.uk/pollutants/).
